# Processing and Characterisation of Banana Leaf Fibre Reinforced Thermoplastic Cassava Starch Composites

**DOI:** 10.3390/polym13091420

**Published:** 2021-04-28

**Authors:** Ridhwan Jumaidin, Nuraliah Ahmad Diah, R. A. Ilyas, Roziela Hanim Alamjuri, Fahmi Asyadi Md Yusof

**Affiliations:** 1 Fakulti Teknologi Kejuruteraan Mekanikal dan Pembuatan, Universiti Teknikal Malaysia Melaka, Hang Tuah Jaya, Durian Tunggal, Melaka 76100, Malaysia; b071710727@student.utem.edu.my; 2 School of Chemical and Energy Engineering, Faculty of Engineering, Universiti Teknologi Malaysia, UTM Johor Bahru 81310, Malaysia; ahmadilyas@utm.my; 3 Centre for Advanced Composite Materials (CACM), Universiti Teknologi Malaysia, UTM Johor Bahru 81310, Malaysia; 4 Faculty of Science and Natural Resources, Universiti Malaysia Sabah, Jalan UMS, Kota Kinabalu 88400, Malaysia; 5 UNIKL MICET, Taboh Naning, Alor Gajah, Melaka 78000, Malaysia; fahmiasyadi@unikl.edu.my

**Keywords:** mechanical, thermal, thermoplastic cassava starch, banana leaf fibre, biocomposite

## Abstract

Increasing environmental concerns have led to greater attention to the development of biodegradable materials. The aim of this paper is to investigate the effect of banana leaf fibre (BLF) on the thermal and mechanical properties of thermoplastic cassava starch (TPCS). The biocomposites were prepared by incorporating 10 to 50 wt.% BLF into the TPCS matrix. The samples were characterised for their thermal and mechanical properties. The results showed that there were significant increments in the tensile and flexural properties of the materials, with the highest strength and modulus values obtained at 40 wt.% BLF content. Thermogravimetric analysis showed that the addition of BLF had increased the thermal stability of the material, indicated by higher-onset decomposition temperature and ash content. Morphological studies through scanning electron microscopy (SEM) exhibited a homogenous distribution of fibres and matrix with good adhesion, which is crucial in improving the mechanical properties of biocomposites. This was also attributed to the strong interaction of intermolecular hydrogen bonds between TPCS and fibre, proven by the FT-IR test that observed the presence of O–H bonding in the biocomposite.

## 1. Introduction

Plastics have increasingly been used as the main product in many sectors, especially in packaging applications, whether as reusable or single-use plastics [1,2]. Globally, plastic production had continually risen for more than five decades. In 2013, plastic production was 299 million tons, and it was anticipated to increase by about 3.9% annually [3]. The plastic demand increased over time due to the low price and convenient use. However, plastics are non-biodegradable and unrecyclable because they are fully made from chemicals such as propylene and ethylene. These factors have been causing the accumulation of plastics and the presence of plastic waste in the environment that have been hugely impacting the ecosystem and humans [4,5,6].

Many studies have been conducted to explore plastic alternatives to overcome these problems. Among the attempts to replace the use of petroleum-based polymers, thermoplastic starch (TPS) has been found as one of the best solutions. TPS has been synthesised from natural starch with the help of a plasticiser and reinforcement for better characteristics; it has good mechanical properties, lower sensitivity towards water, gas barrier, good thermal stability, low density, and also low cost. Most importantly, the TPS material can be easily degraded in the soil, which means that it is environmentally friendly [7]. For the reinforcement, natural fibres were used that are obtained from plants and animals. These natural fibres are also eco-friendly and have good mechanical properties; hence, incorporation with a plasticiser is useful in improving the characteristics of the TPS material [8,9]. Various types of natural fibres have been used as reinforcement in polymer composites, including ginger [10,11], kenaf [12,13,14], flax [15], hemp [16], kapok [17], wood [18], water hyacinth [19], cotton [20], sugarcane [21,22,23], corn [24], ramie [25], and sisal [26], as well as sugar palm [27,28,29,30,31,32,33,34,35,36]. In addition, beeswax is also known as a good matrix because it is able to reduce the hydrophobicity of starch, which consequently improves processability [37]. In order to obtain this TPS material, the starch is processed by hot compression moulding together with the plasticiser and the fibre reinforcement [7].

**Figure 1:**
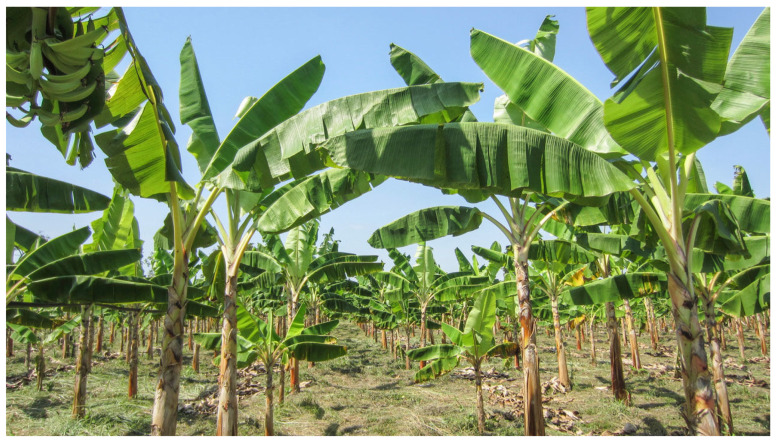
Banana *Musa sp.* plantation.

Some horticulturists believe that bananas were the first fruit on earth [38]. Their origin is placed in Southeast Asia, in the jungles of Malaysia, Indonesia, or the Philippines, where many varieties of wild bananas are still available today [39]. Bananas are grown in more than 150 countries, and 105 million tonnes of fruit are produced each year. Bananas that are cultivated for local consumption are generally planted in extensive, traditional systems. Banana plants are often mistaken for trees or palms—they are actually herbs [40]. The banana is a perennial plant that replaces itself. Bananas do not grow from a seed, but a bulb or rhizome, and it takes 9 to 12 months from sowing a banana bulb to harvesting the fruit. Figure 1 shows a banana plantation. Currently, the banana leaves are discarded and only a small portion are sold for traditional food packaging; meanwhile, the use for other applications is somehow limited [41]. Banana plants are easy to grow; therefore, all parts of the plants are readily available over the year, including the leaf. Almost 480 kg of leaves, 3 tons of pseudostem, 440 kg of skins, and 160 kg of stalks wastes are produced for every ton of banana plants harvested [42]. Due to this tonnage of wastes produced, many efforts have been taken to reduce the waste by averting disposal and converting them to other functions. Some studies were performed to analyse the capability of the natural fibre-reinforced composite. A study by Bilba et al. [43] used banana leaf fibre (BLF) as the reinforcement of a composite material, which revealed good improvement in the mechanical properties of the produced composite. The material was also found to degrade under natural environmental conditions. Another study by Mo et al. [44] used thermoplastic cassava starch and cellulose fibre and proved that the modification of thermoplastic starch had improved the properties of the material. The moisture absorption had reduced, while the mechanical and thermal properties had increased. Hence, the incorporation of TPS with banana leaf fibre reinforcement was used in this study to analyse the effect of this fibre on the mechanical and thermal properties of the composite.

Even though there are studies reporting on using BLF in composites [45,46,47], none were found on the utilisation of BLF in a TPS matrix. Thus, further study was done to investigate and enhance the thermal and mechanical properties of thermoplastic cassava starch reinforced with banana leaf fibre. In addition, this study was also carried out to produce material that is biodegradable and recyclable, and therefore capable of pollution reduction and is greener to the environment in terms of biodegradability and renewability.

## 2. Materials and Methodology

### 2.1. Materials

Banana leaves were collected from the banana field at Kota, Negeri Sembilan, Malaysia. The banana leaf fibre (BLF) was extracted by using a water retting process for 4 weeks of immersion. The retted leaves were washed in running water, and the fibre was removed and then dried at 100 °C for 5 h. The dried fibre was cut into 5 mm length and stored in a zip-locked plastic bag until further use. Food-grade cassava starch was procured from Antik Sempurna Sdn. Bhd, analytical grade glycerol was purchased from QRec Chemicals, and analytical grade refined beeswax was purchased from Sigma Aldrich.

### 2.2. Samples Preparation

Thermoplastic cassava starch (TPCS) was prepared by a mixture of 63 wt.% starch, 27 wt.% glycerol, and 10 wt.% beeswax. This mixture was blended by using a BL1515 Dry Mixer from Khind (Shah Alam, Selangor, Malaysia) for 5 min and thermo-pressed at 155 °C for 1 h using a GT7014-P30 C Plastic Hydraulic Molding Press from GOTECH Testing Inc (Taichung City, Taiwan). A similar process was carried out for the preparation of TPCS/BLF composites by varying the BLF content from 10 to 50 wt.% in the TPCS matrix. The prepared samples were stored immediately in a desiccator containing silica gel prior to the conditioning process in order to avoid unpredicted moisture absorption.

### 2.3. Thermogravimetric Analysis (TGA)

A TGA test was carried out using a Mettler-Toledo AG analyser (Greifensee, Switzerland). The test was performed in a temperature range between 25 and 800 °C at a heating rate of 10 °C/min under a dynamic nitrogen atmosphere. A sample of 5–10 mg of the composite was heated in an alumina crucible pan.

### 2.4. Mechanical Tests

Tensile and flexural tests were conducted using the Instron 5969 (Norwood, MA, USA) machine according to ASTM D638 and ASTM D790, respectively. Specimens of tensile were cut with dimensions of 65 mm (L) × 6 mm (W) × 3 mm (T), while flexural specimens were cut with dimensions of 130 mm (L) × 13 mm (W) × 3 mm (T). Five specimens were tested with a crosshead speed of 5 mm/min for tensile strength, while flexural strength was tested at 2 mm/min, both under room temperature of 23 ± 1 °C and relative humidity of 50 ± 5%.

### 2.5. Fourier Transform Infrared Spectroscopy (FT-IR)

FT-IR spectroscopy was used to detect the functional groups and chemical characteristics of the material. The spectra were obtained using Jasco FT-IR6600 (Maryland, United States). The specimens used were in the measurement of 1 mm (L) × 1 mm (W) × 3 mm (T). The spectra of the samples were collected within a range of 4000 to 400 cm^−1^.

### 2.6. Scanning Electron Microscopy

SEM micrographs from the fractured tensile test samples were obtained using Zeiss Evo 18 (Jena, Germany) with 10 kV acceleration voltage. The fractured part of the samples was cut and gold-coated over the surface uniformly before running the test.

### 2.7. Statistical Analysis

SPSS software was used to perform the analysis of variance (ANOVA) on the obtained experimental results. Tukey’s test was employed to conduct means comparisons at a 0.05 level of significance (*p* ≤ 0.05).

## 3. Results and Discussion

### 3.1. Thermal Properties

According to Blasio et al. [48], thermogravimetric analysis (TGA) or thermogravimetry (TG) is a process in which the mass of a polymer is evaluated as a function of time or temperature in a controlled atmosphere while the sample is subjected to a controlled temperature program. The range of temperature is generally from ambient to 1000 °C for polymer applications. The thermal degradation of the composite can be observed as weight loss with respect to temperature [49].

**Figure 2:**
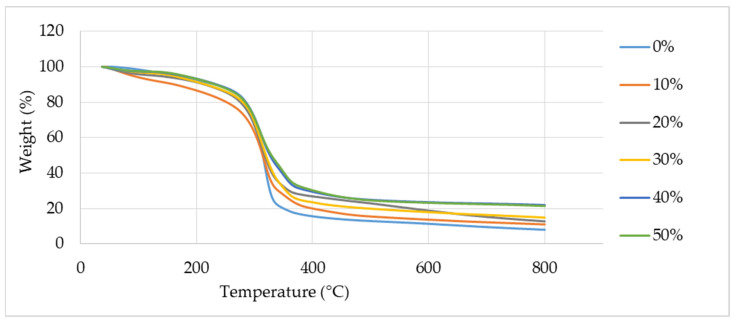
TGA curve of thermoplastic cassava starch (TPCS) + banana leaf fibre (BLF).

Figure 2 indicates the TG curves of the TPCS reinforced with banana leaf fibre from 0 to 50 wt.% fibre content. It was observed that the sample’s weight was reduced due to degradation as the temperature was raised. At temperatures below 200 °C, the first phase of degradation occurred. This might be attributed to the evaporation of moisture from water present in the sample. A similar finding was observed by Sahari et al. [50] on thermoplastic sugar palm starch composites. A slight degradation had occurred at a temperature of 168 °C due to water removal from the sample and resulted in a reduction in weight. Next, the weight loss between the temperatures of 150 and 380 °C for TPCS reinforced with banana leaf fibre was associated with the decomposition of natural fibre’s major components; cellulose, hemicellulose, and lignin [50]. The decomposition of cellulose and hemicellulose took place at temperatures between 200 and 270 °C, while lignin and the final decomposition of cellulose occurred in the range of 270 to 370 °C [51]. Meanwhile, maximum starch decomposition was observed at 300 °C, which might be ascribed to the degradation of starch along with the fibre, and the residue formed after the degradation required higher temperatures for subsequent degradation [49]. This finding was in agreement with a study by Edhirej et al. [52] on the thermal behaviour of sugar palm fiber/cassava bagasse reinforced with cassava starch hybrid biocomposites. The first weight loss of the biocomposites was due to moisture evaporation. Then, the decomposition of hemicellulose, cellulose, and lignin took place between 150 and 380 °C.

It was found that the weight reduction in the sample slowed down as the fibre contents were increased from 0 to 50 wt.%. This finding was in parallel with a study conducted by Wattanakornsiri and Tongnunui [53], who stated that the weight loss of the composites was gradually reduced as the fibre content was increased and the degradation temperatures were increased when fibres existed in the composites. These were attributed to the higher thermal stability of fibres compared to the starch, and the strong compatibility of both polysaccharides. The addition of fibres influenced the increase in thermal resistance of pure TPS due to the good interaction between the matrix and the fibre.

**Figure 3:**
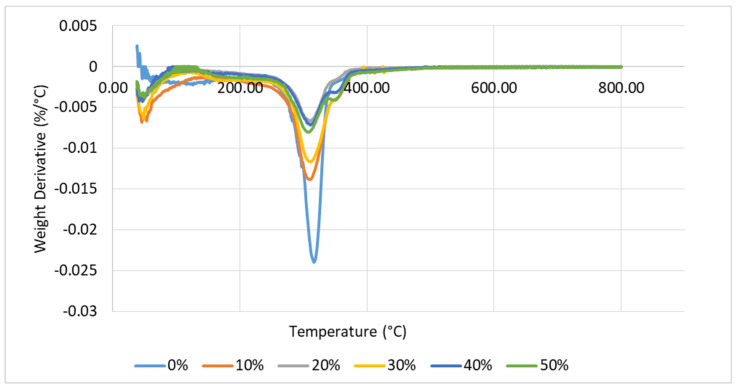
DTG curve of TPCS-banana leaf fibre.

Meanwhile, Figure 3 shows the derivative thermogravimetry (DTG) curve for TPCS with banana leaf fibre reinforcement. The maximum DTG peak occurred at a temperature range between 200 and 400 °C. However, the DTG peaks were lowered as the fibre was added and showed very small variations [52]. Due to the degradation of fibre, starch, and glycerol that produced volatile substances including carbon monoxide and carbon dioxide, a rapid weight loss was observed in this stage [54]. According to Monteiro et al. [55], the first stage of DTG peak was associated with moisture reduction. The degradation of fibre components started at the second stage, which was the main DTG peak. The peak represented cellulose decomposition, while hemicellulose and lignin decompositions took place at the shoulder peak and tail peak, respectively. The remaining weight could be attributed to the char from the decomposition reactions. This showed that the DTG curve gave a better understanding of the composite material’s thermal behaviour.

This finding is in agreement with a study performed by Tajvidi and Takemura [56] on the thermal degradation of natural fibre reinforced with polypropylene (PP) composites, which were kenaf fibre and woof flour composites. Kenaf fibre and wood flour composites possessed high decomposition rates that occurred in three stages. The first stage took place at temperatures between 250 and 300 °C, which was attributed to hemicellulose and PP decomposition. At a temperature range of 300 to 400 °C, the second stage occurred due to cellulose decomposition and the final decomposition was observed around 450 °C.

### 3.2. Mechanical Properties

#### 3.2.1. Tensile Test

The tensile test is a test performed to analyse the tensile strength and modulus. The amount of force used to break the specimen and the extension of the specimen is measured by using this test [27,33,57]. The tensile strength is a key parameter in the analysis of the material’s structure and is obtained from the ultimate tensile strength (UTS) data [19,23,58,59,60]. The tension was applied to the sample until the material fractured and the force was recorded [61]. The differences in the composition of thermoplastic cassava starch (TPCS) initiated the change in the mechanical properties of TPCS as well.

**Figure 4:**
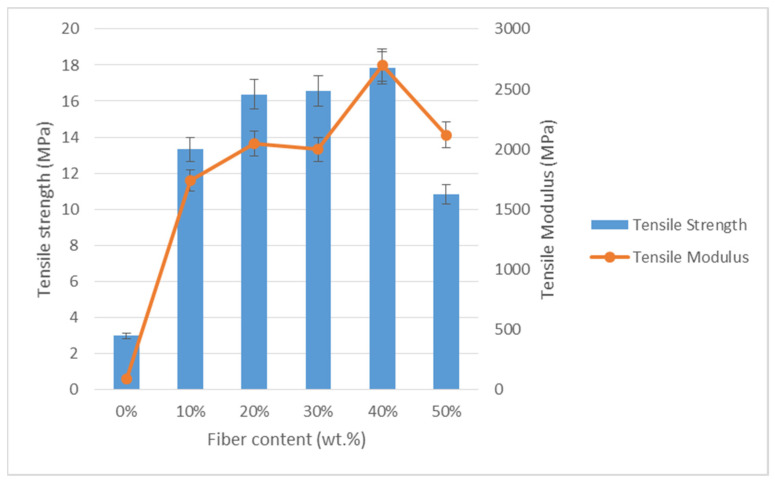
Tensile strength and modulus of TPCS/BLF composites.

Figure 4 indicates the tensile properties of TPCS/banana leaf fibre composites, which are tensile strength and modulus, respectively. Table 1 shows the analysis of variance (ANOVA) of the tensile properties. There was a statistically significant difference between the mean tensile strength, modulus, and elongation from one level of composites to another, because the *p*-value was less than 0.05. As different percentages of fibre were added, it was noticed that the tensile strength and modulus tended to become higher when the banana leaf fibre’s contents were increased. Based on Figure 4, there was a significant increment from 0 to 30 wt.% fibre content, while the highest strength was obtained from the TPCS added with 40 wt.% banana leaf fibre with the values of 2.96, 13.31, 16.37, 16.56, and 17.85 MPa, respectively. This showed that the optimum amount of banana leaf fibre needed as reinforcement was 40 wt.%.

The mechanical properties of reinforced composite materials depend on the properties of the matrix, amount of reinforcement, and mutual interfacial wettability [62]. The addition of fibre led to significant improvement in tensile modulus and strength of the composites due to the strong combination of polysaccharides, cellulose fibres, and starch [63]. According to Jumaidin et al. [64], the tensile strength involves great interfacial matrix–fibre adhesion that can be attributed to strong bonding between them. Thus, it is possible to achieve a good stress transfer from the matrix to the fibre. This finding agreed with the SEM testing result that showed good interfacial adhesion between the matrix and the fibre. Besides that, the amylose content in cassava starch and the quantity of plasticiser also influenced the properties of thermoplastic starch, and thus contributed to higher tensile strength and rigidity along with low ductility. A similar finding was observed in a study by Chotiprayon [65] on the mechanical properties of TPCS/PLA reinforced with coir fibre (CF). The tensile strength and modulus were improved with increasing CF fibre content due to the reinforcing effect of the CF fibre.

However, for higher than 40 wt.% of banana leaf fibre content (i.e., 50 wt.%), the tensile strength was found to decrease. At this point, the reduction in strength might be due to the higher content of fibre that caused the fibre to agglomerate within the matrix. A previous study also showed the same trend of results, where the tensile strengths of hybrid composite films were increased when the SPF contents were raised from 2%, 4%, to 6%, but the strength was reduced at 8% of SPF [66]. According to El-Shekeil [67] and Ayu et al. [68], the fibres were not adequately wetted by the matrix, and the higher content of fibre caused the agglomeration and blocking of stress transfer from the matrix to the fibre. This finding was in agreement with the SEM result, which showed that the matrix and the fibre were not distributed evenly, and rough cleavage fracture was observed as the fibre content reached 50 wt.%.

Tensile modulus was obtained from the initial slope and the linear region of stress–strain curves [69]. For the tensile modulus, the finding showed the same trend of increasing modulus from 0 to 40 wt.% but slight decrement at 30 wt.% of fibre loading. Edhirej et al. [66] stated that tensile modulus determines the stiffness of a material, which indicates that higher tensile modulus results in stiffer composite material. The higher content of fibre loading leads to a significant increment of tensile modulus. Hence, from the study, the result showed that the addition of banana leaf fibre also resulted in the increment of the tensile modulus.

However, the tensile modulus decreased at 50 wt.% of fibre, just like the tensile strength. This finding trend was due to the high amount of fibre that had a low strain [67]. This also might be due to structural changes in the arrangement of starch that occurred when fibres were added, making the matrix less compact [66]. This finding was in agreement with a study conducted by Cheng et al. [70] on the properties of a lignocellulosic fibre (LCF)/CaCO3 (CG) hybrid with TPS (0, 27, 54, and 81 g). As the dosage of LCF/CG hybrid increased to 54 g, the tensile strength and modulus were increased, but then declined when the dosage reached 81 g. This outcome was attributed to the poor reinforcing effect by the fibre when the amount of fibre used was too high, which generally increased the strength but led to decreased toughness. The decreasing tensile modulus might also be attributed to the agglomeration of fibres as higher fibre contents were loaded [65].

**Figure 5:**
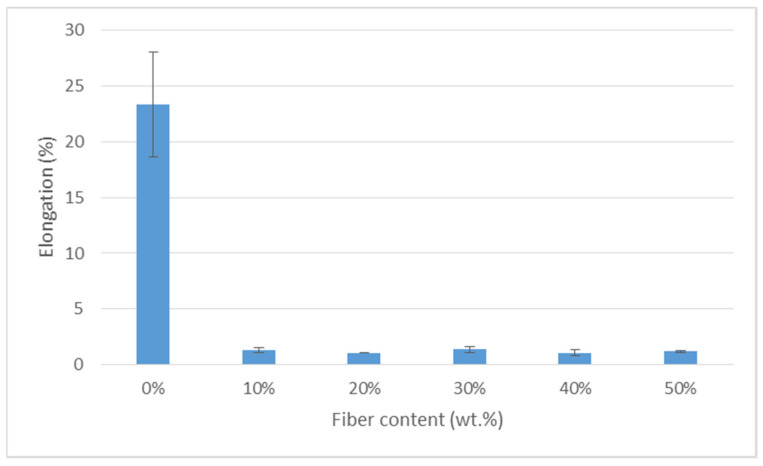
Elongation at break of TPCS/BLF composites.

Figure 5 demonstrates the elongation at break of the TPCS/BLF composites. Analysis of variance (ANOVA) of the elongation value showed that there was a statistically significant difference (*p* < 0.05) between the elongation value of the TPCS matrix and the composites. In general, the elongation of the samples showed significant decrement following the incorporation of BLF. For the elongation at break, the finding showed a decreasing trend of increasing loading from 0 to 50 wt.%. Likewise, contrary to the increase in tensile strength and tensile modulus, the elongation at break for the biocomposite decreased from 0.31% to 0.01% as the amount of banana leaf fibre increased from 0 to 50 wt.% in the cassava TPS. The higher content of fibre loading had led to a significant decrement of elongation at break. Hence, from the study, the result showed that the addition of banana leaf fibre also resulted in the decrement of the elongation at break. This trend was due to the high amount of fibre [57]. The result gained was in agreement with a study conducted by Sanyang et al. [71] on the elongation properties of sugar palm cellulose (SPC) with TPS ((0%, 1%, 3%, 5%, 10%). According to Sanyang et al. [71], the elongation at break for the composite films reduced from 40.99% to 32.8% as the SPC loading increased from 1 to 10 wt.% in the neat SPS films. Moreover, this finding was in agreement with the tensile strength behaviour of the composites, which showed a significant increment in the value with the addition of fibre. This might be associated with higher resistance to deformation following the increment of material strength; hence, decreasing the elongation of the material. Similar findings on the decrement of elongation following the addition of fibre were also reported in previous studies [64]. Moreover, the incorporation and increase in banana leaf fibre decreased the molecular mobility of the cassava biopolymer matrix, which made the biocomposite materials stiffer [33,57]. Hence, banana leaf fibre-reinforced cassava biocomposite films became more resistant to break, stiffer, and less stretchable compared to the neat TPS.

#### 3.2.2. Flexural Test

A flexural test was performed to obtain the flexural strength and modulus of the composite. The flexural strength is based on the combination of compressive and tensile strengths to observe the bending strength of the composite materials [61]. The interfacial shear strengths that existed between the matrix and fibre also agreed with Azammi et al. [72].

**Figure 6:**
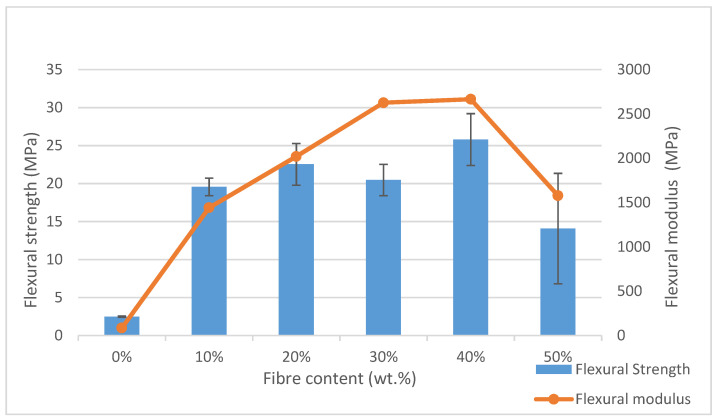
Flexural strength of TPCS + banana leaf fibre.

Figure 6 indicates the flexural strength and flexural modulus. Generally, the outcome of this flexural test exhibited similar trends with the tensile test. Table 2 shows the analysis of variance (ANOVA) of the flexural properties. The *p*-value was less than 0.05; therefore, there was a statistically significant difference between the mean flexural strength and modulus from one level of composites to another. Both flexural strength and modulus of the composites were significantly increased (*p* < 0.05) with increasing banana leaf fibre content. Figure 6 shows that the flexural strength increased from 0 to 20 wt.% but slightly decreased at 30 wt.%, while the highest strength was obtained at 40 wt.% banana leaf fibre before starting to decrease.

Similar factors that were mentioned in the tensile test could also be attributed to the improvement of the flexural properties of the composites. This finding was associated with the addition of fibre as reinforcement that contributed to great interfacial adhesion as good stress transfer between the matrix and fibre occurred [73]. The fibres acted as load carriers and stress was transferred from the matrix along the fibres that led to uniform and efficient stress distribution, resulting in better mechanical properties [74]. A similar finding was observed in a study by Jumaidin et al. [75], where the flexural strength of a TPSA/seaweed composite was observed to increase until 30 wt.% of seaweed content, and then reduced at 40 wt.%. According to Sahari et al. [50], it was considered a normal outcome when the flexural strength and modulus were increased as fibre was added to the matrix. However, the flexural strength was decreased at 50 wt.% of fibre, which might be attributable to the weak fibre distribution and non-uniform interfacial bonding between the fibre and matrix [76]. Another similar finding was observed in a study by Shinde et al. [8] on the properties of coir fibre-reinforced polypropylene composite. The flexural strength of the composite had increased up to 60 wt.% fibre content and started to decrease as higher fibre content was loaded. This might be attributable to the insufficient matrix to cover all the surfaces of the fibre.

Meanwhile, the flexural modulus was used as a stiffness indicator of the material [77]. Based on Figure 6, the flexural modulus was also increased from 0 to 30 wt.% and peaked at 40 wt.% of fibre content. The increment of the flexural modulus was also attributed to similar justifications in the tensile test results. The increase in the flexural modulus might be due to the improvement of the fibre/matrix interaction [78]. According to Elanchezhian et al. [79], higher stress was required for the same deformation when a higher amount of fibre was added. Thus, the incorporation of the fibre into the matrix resulted in an increase in the flexural modulus.

Moreover, the reduction in modulus at 50 wt.% of fibre content might be caused by the insufficient amount of matrix to cover all the banana leaf fibre surface [8]. This finding was in agreement with a study conducted by Hassan et al. [54], who reported improvements in flexural strength and modulus following the addition of PLA to starch/cellulose foam composites until 4.86% PLA but started to decrease when the addition of PLA was higher than 4.86%. Another study by Jumaidin et al. [64] on TPCS/CGF biocomposite also reported a similar finding in which the flexural strength and modulus were improved due to the incorporation of cogon grass fibre. The biocomposite became less ductile compared to the TPCS matrix due to the strong bonding between the matrix and fibre.

### 3.3. Samples Characterisation

#### 3.3.1. FT-IR Test

FT-IR spectroscopy is a technique of measuring the frequencies and intensities that a sample absorbs based on the interaction between the IR radiation and samples that can be in the solid, gaseous, or liquid state [80]. These frequencies detect the chemical functional groups that are responsible for the radiation absorption at different frequencies [81,82,83,84]. FTIR testing was carried out to investigate the effect of reinforcing agents’ addition on the thermoplastic starch. This also functioned to analyse the interaction between starch, fibre, and glycerol [53].

**Figure 7:**
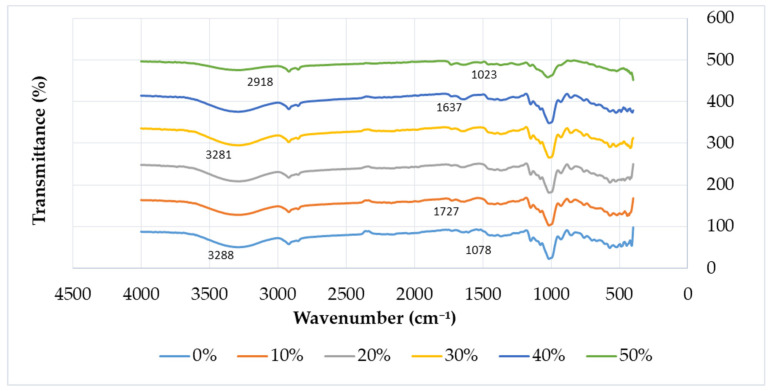
FT-IR spectra of TPCS + banana leaf fibre.

Figure 7 displays the FT-IR data for TPCS reinforced with banana leaf fibre composites from 0 to 50 wt.% of fibre content. In general, all the spectra of TPCS composites showed the same pattern of bands. This finding exhibited that the amount of banana leaf fibre added to the composites did not change or chemically affect the TPCS. A similar finding was reported with the study by Sanjay et al. [85], who reported that the native sugar palm starch (SPS) had a similar spectrum with the agar spectrum. The stronger interaction between the components of the polymer blends can be identified at a lower wavenumber.

Based on Figure 7, both pure TPCS and TPCS with banana leaf reinforcement displayed the same broad bands from 3200 to 3500 cm^−1^, which indicated the presence of O–H groups due to the hydroxyl groups found in lignin, cellulose, and hemicellulose in the banana fibre [75]. This finding showed that the starch had high sensitivity towards water molecules due to the presence of hydroxyl groups, and was also attributed to the hydroxyl groups stretching due to hydrogen bonding between the molecules [50,57]. However, it was noticeable that the O–H bond peak shifted to a lower wavenumber when the fibre content was increased. Specimen with 0% fibre peaked at 3288 cm^−1^, while specimen with 30% fibre peaked at 3281 cm^−1^. A similar O–H trend was found in a previous study performed by Prachayawarakorn et al. [86] for both TPCS/kapok fibre and TPCS/jute fibre. The O–H groups that appeared at 3300 to 3500 cm^−1^ were slightly shifted to lower wavenumbers, which was attributed to the free, intra-, and intermolecular bound hydroxyl groups. This finding revealed that the formation of new hydrogen bonds between the TPCS matrix and fibre existed. Thomas et al. [87] reported a similar finding by indicating the presence of a hydrophilic O–H group at 3300 cm^−1^ which proved the hygroscopic nature of cellulose.

Meanwhile, the presence of C–H stretching was recorded with the strong band within the range of 2850–3000 cm^−1^ due to the vibrations of cellulose [87]. This C–H band also corresponded to the natural fibre components, which were cellulose and hemicellulose [51]. Other than that, the presence of anhydroglucose ring C–O stretching was found in the range of 1000–1200 cm^−1^. This band was also attributed to the stretching of the C–O group from lignin in the natural fibre [51]. This finding was in agreement with a study by Hassan et al. (2019), who also recorded a similar outcome with symmetric and asymmetric C–H stretching vibrations at 2849 and 2917 cm^−1^, respectively, and the presence of C–O stretching between 980 and 1160 cm^−1^ in TPS/cellulose composite reinforced with PLA. Another study by Jumaidin et al. [64] also showed C–H stretching vibrations at 2900 cm^−1^ in cellulose and hemicellulose, and also resembled the vibration from CH_2_ and/or CH_3_ in a TPCS/CGF composite.

The presence of C=O stretching can be found in the range of 1630–1740 cm^−1^. However, this C=O peak could only be found in TPCS reinforced with banana leaf fibre samples and was absent in the pure TPCS sample. According to Sgriccia et al. [88], who obtained a similar outcome with the kenaf and hemp fibre composites, the presence of C=O stretching might be attributed to the presence of hemicellulose in the fibre. Another related finding in a study by Lomelí-Ramírez et al. [89] indicated the presence of carbonyl group vibration (C=O) at 1720 cm^−1^ in biocomposites of cassava starch with green coconut fibre reinforcement, and was absent in the native starch sample.

#### 3.3.2. SEM Test

**Figure 8:**
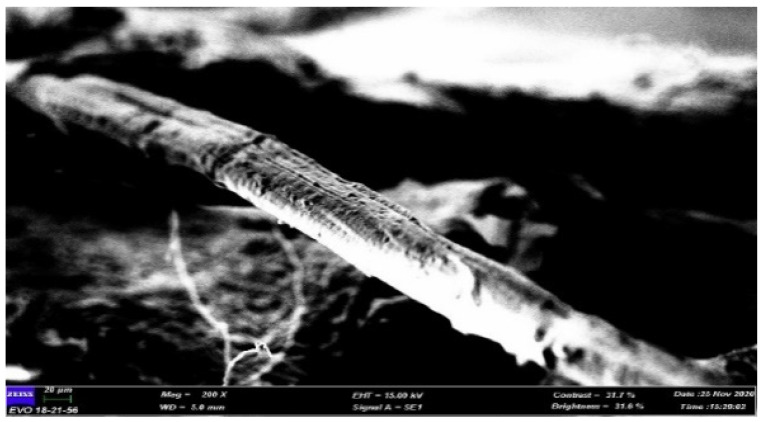
SEM micrograph of banana leaf fibre.

**Figure 9:**
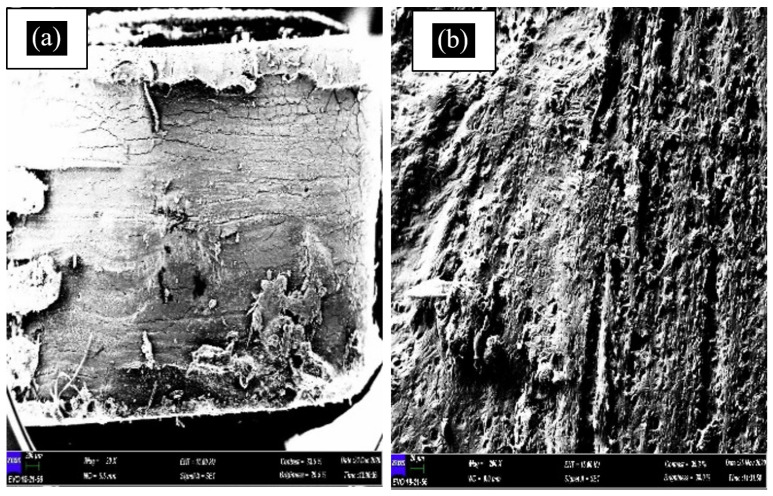
SEM micrograph of pure TPCS (**a**,**b**).

**Figure 10:**
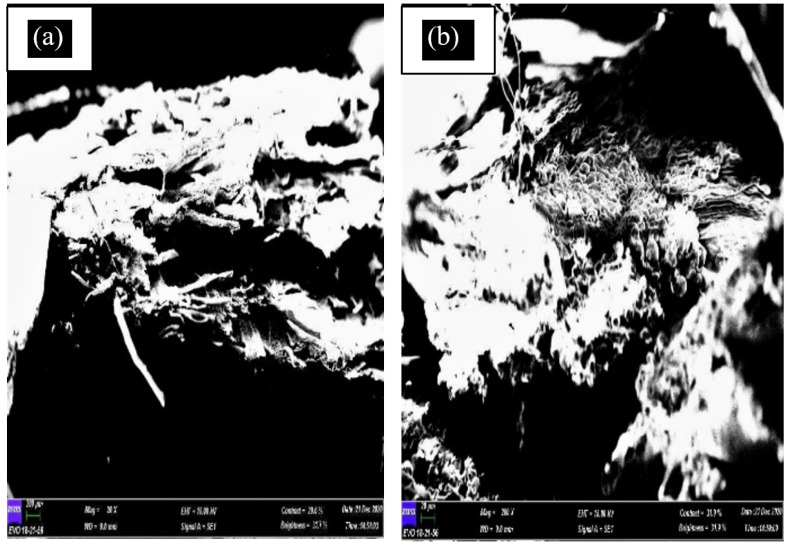
SEM micrograph of TPCS + 50 wt.% banana leaf fibre (**a**,**b**).

**Figure 11:**
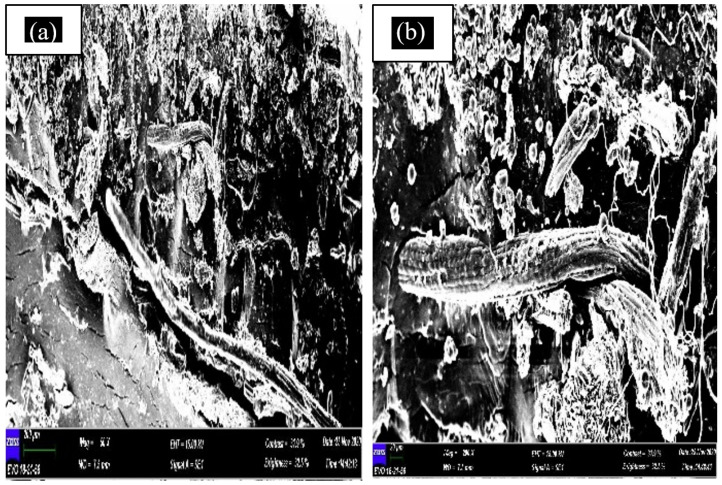
SEM micrograph of TPCS + 10 wt.% banana leaf fibre (**a**) and the breakage of the fibre can be observed more clearly, and a porous surface appeared (**b**).

**Figure 12:**
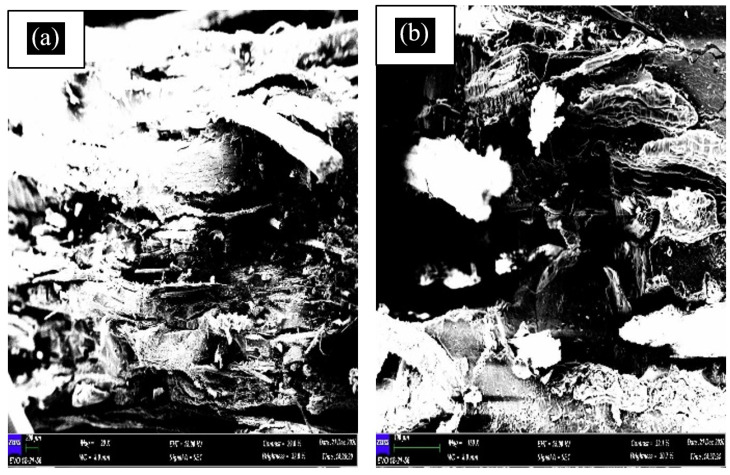
SEM micrograph of TPCS + 30 wt.% banana leaf fibre; (**a**) the evidence of fibre break and (**b**) more void on fracture surface.

A scanning electron microscope (SEM) is the instrument used to examine and analyse microstructure morphology. By performing this test, the failure morphology of tensile fractured samples can be observed. This SEM morphological research also can be used to analyse the mechanical properties of materials and the interfacial adhesion between the matrix and fibre. Figure 8 shows the SEM micrograph of banana leaf fibre, while Figure 9, Figure 10, Figure 11 and Figure 12 exhibit the microstructures of the fractured surface of TPCS reinforced with banana leaf fibre composite with different fibre contents (0, 10, 30, and 50 wt.%). The findings showed a variation of the microstructures of each specimen when different fibre contents were loaded. The fibre breakage could be found in all composites as a result of tensile fracture due to good stress-transfer from the matrix to fibre that gave reinforcement effect to the composites [51]. This finding was in parallel with tensile test results which showed increments in tensile strength and modulus.

For the specimen with 0 wt.% fibre content (Figure 9), only the matrix which consisted of TPCS was observable. No remaining starch granules were observed, which might be associated with the good shear force induced by glycerol addition that led to great plasticiser dispersion [90]. This can be seen in both Figure 9a,b of the sample surface. According to Jumaidin et al. [91], the starch granules should be broken down to form a continuous phase with glycerol in thermoplastic starch. This finding showed that the melt mixing of starch and glycerol had enhanced the plasticisation of starch. However, it was noticeable that the surface of the specimen appeared to be in granular shape and a bit shiny.

Next, for the specimen with 10 wt.% banana leaf fibre reinforcement, it was observed that no phase separation occurred because there was good adhesion between the matrix and fibre (Figure 10). The compatibility of the matrix and the fibre was considered high, due to good fibre wetting by the matrix to form a homogenous surface when combined with the fibre [8]. Both Figure 10a,b shows the evidence of fibre break on the fracture surface. A study conducted by Mueangta et al. [86] indicated a comparable finding in which both TPCS/kapok fibre and TPCS/jute fibre showed better compatibility between the matrix and fibre. This finding was attributed to the surface wetting on both kapok and jute fibres by the TPCS matrix. Moreover, the surface of the fibre appeared to be covered by TPCS and the breakage of the fibre could be clearly seen. This finding might be ascribed to the strong intermolecular hydrogen bonds between the TPCS and the fibre [44].

The increment of fibre content influenced the structure of the composite, as observed when the fibre content was raised to 30 wt.%. Figure 11a indicates that the banana leaf fibre was still covered by the matrix, while in Figure 11b, the breakage of the fibre can be observed more clearly, and a porous surface appeared. A similar finding was obtained in a study carried out by Mo et al. [44] on the properties of TPCS reinforced with banana fibre. The findings indicated that the surface of banana fibre was coated by TPS and the fibre breakage can be observed clearly. This was associated with the strong intermolecular hydrogen bonds between TPS and banana fibre. This finding was also correlated with the increment of tensile strength, which proved that TPS was suitable as the matrix of natural fibre.

At 50 wt.% fibre content, it can be observed that the matrix and fibre were not evenly distributed (Figure 12). More rough fracture was observed at higher fibre content than lower ones. Figure 12a shows the evidence of fibre break while Figure 12b shows the presence of more void on fracture surface. This finding can be ascribed to the insufficient dispersion due to the increased fibre content; the agglomeration caused poor matrix–fibre interactions thus resulting in poor interfacial adhesion [44]. The fibre content was limited by insufficient dispersion or high viscosity during the process. The interaction among the fibre will form the aggregates, and this will induce defects in the matrix which affect the structure of the composites [52].

## 4. Conclusions

In this study, novel biocomposites derived from thermoplastic cassava starch and BLF were produced, and their thermal, mechanical, and morphological properties were investigated. It was found that the addition of BLF increased the thermal stability of the material, indicated by the higher decomposition temperature of the composites than the neat TPCS. Significant improvements in the tensile and flexural properties of the material were evidence by the addition of BLF. The morphological investigation on tensile fracture revealed fibre breakage phenomena, which indicated good fibre–matrix bonding and reinforcement in the material. Samples with higher BLF content at 50 wt.% showed a decrement in the mechanical properties. Overall, both the thermal and mechanical properties of this TPCS were improved with the addition of BLF. Hence, this study revealed that BLF has the potential to be used as natural reinforcement material to a bio-based polymer matrix.

## Figures and Tables

**Table 1:** ANOVA of tensile properties.

Variables	Tensile Strength	Tensile Modulus	Elongation
Mixture	0.001 *	0.00 *	0.000
Sum of Squares	447.381	11,811,337.04	0.227
df	5	5	5
Mean Square	89.476	2,362,267.407	0.45
F	9.526	23.894	31.412

* Note: Significantly different at *p* ≤ 0.05.

**Table 2:** ANOVA of flexural properties.

Variables	Flexural Strength	Flexural Modulus
Mixture	0.00 *	0.00 *
Sum of Square	1032.913	13,717,646.16
*df*	5	5
Mean Square	206.583	2,743,529.231
F	15.973	37.269

* Note: Significantly difference at *p* ≤ 0.05.

## Data Availability

This study did not report any data.
